# Does the Coronavirus Epidemic Take Advantage of Human Optimism Bias?

**DOI:** 10.3389/fpsyg.2020.02001

**Published:** 2020-08-26

**Authors:** Hugo Bottemanne, Orphée Morlaàs, Philippe Fossati, Liane Schmidt

**Affiliations:** ^1^Control-Interoception-Attention team, Institut du Cerveau (Paris Brain Institute, ICM), UMR 7225/UMR_S 1127, Sorbonne University/CNRS/INSERM, Paris, France; ^2^Department of Adult Psychiatry, Pitié-Salpêtrière Hospital, Assistance Publique-Hôpitaux de Paris (AP-HP), Paris, France

**Keywords:** coronavirus, COVID-19, belief, perceived risk, predictive coding, cognitive biases, bayesian brain

## Introduction

On March 15, the SARS-Cov2 outbreak had affected more than 160,000 people worldwide and 6,000 people had died. While the World Health Organization had declared a public health emergency as early as the 30th of January, and subsequent evidence had highlighted the threat of a global pandemic, it took many more weeks for many governments and individuals to put into place and adopt precautionary measures. Some observers were therefore surprised about the discrepancy between the available official warnings and individual perceptions of the risks associated with COVID-19.

When confronted with novel pathogens, humans generate several beliefs about the short- and long-term consequences of the new threat. For example, they estimate their likelihood of being exposed, getting infected or infecting others, but also the severity of the emerging event, the controllability of the epidemic, and the efficiency of psychological and physiological coping responses (Rogers, 1975). Numerous studies have shown that protective measures—in terms of containment or body protection—are largely dependent upon individuals' ability to accurately perceive these consequences (Rogers, 1975). Due to the absence of treatment and vaccination for COVID-19, the success of these measures is particularly critical.

Building on evidence from past epidemics and three decades of research in psychology suggesting that various cognitive biases influence beliefs about life hazards, we propose that such cognitive biases have contributed to the discrepancy between early warnings about the danger of SARS-CoV-2 and slow growth of consideration for these warnings. Specifically, we focus on belief updating biases, which result from the way the human brain creates and updates beliefs about the world in the face of evidence. We critically discuss this idea with respect to other types of cognitive biases such as oversensitivity to rare events, cognitive myopia, and risk aversion.

## Belief Formation, Cognitive Bias, and The Brain

Our mind is woven by our beliefs about the world. At every moment, our beliefs define what we expect, what we perceive and what we choose. An influential theory, known as the Bayesian brain and predictive coding theory, proposes that our brain continuously develops predictive models about its environment (Friston et al., 2017). These models are compared to incoming sensory evidence in order to generate inferences, which are in turn used to filter perception and guide our actions.

When our brain detects a difference between what it predicts and what it perceives, it generates a prediction error that is then used to update the predictive model. The brain thus continuously learns from changes in its environment, thereby improving its predictions of the world (Moutsiana et al., 2015). The predictive coding theory has initially been proposed as a mechanism for the way in which the brain processes sensory information in the visual cortex (Friston et al., 2017). Since then, it has been applied in various fields of research, and offers a plausible framework for explaining how the brain creates our mental world through perpetual cycles of inferences (i.e., we perceive and act according to our beliefs) and updates (i.e., we modify our beliefs according to what we perceive, what we do, and how our actions affect our perceived environment) (Friston et al., 2017).

However, the cycle between predictions and their update is often asymmetrical. Research has shown that positive illusions about control, superiority and unrealistic optimism [see Jefferson et al. (2017) for review] can determine beliefs about health, romantic relationships, or professional success (Scheier and Carver, 2008). Unrealistic optimism is defined by a general tendency to overestimate the probability of experiencing positive life events, and to underestimate the probability of experiencing adverse life events compared to a similar other person's risk (Weinstein, 1980; Weinstein et al., 2005). While unrealistic optimism is one of the most robust phenomena in human psychology, many have challenged its existence by highlighting confounds such as ceiling or flooring effects, regression to the mean and under-estimation of rare events. All of those effects can indeed obscure the phenomenon when being inferred solely on a group level (Harris and Hahn, 2011). Others consider that unrealistic optimism is a personality trait rather than a cognitive bias, and as such is most frequently expressed in specific groups of people such as smokers or gamblers (Shah et al., 2016).

However, more recent research from cognitive and computational neuroscience is using a novel approach to understand such biased prospective beliefs: it assesses on the individual level how much a person changes their belief about the probability of experiencing adverse life events (Moutsiana et al., 2015). These studies show that such belief updating is “optimistically” biased because favorable information is more considered than unfavorable information, which tends to be neglected (Sharot et al., 2011; Moutsiana et al., 2015; Kuzmanovic and Rigoux, 2017). This valence-dependent bias influences beliefs about the future, but also beliefs about oneself and the world (Sharot et al., 2011). Importantly, this line of research has re-validated the concept of unrealistic optimism as a cognitive bias in human judgement and decision-making by providing a mathematical formalization and a neurobiological basis for it (Moutsiana et al., 2015; Kuzmanovic and Rigoux, 2017).

## Has Unrealistic Optimism Hindered Risk Awareness Associated With the Spread of COVID-19?

In January, when the Chinese city of Wuhan was quarantined, there were only a few cases detected in Europe and the USA. An increasingly dramatic contrast grew between the rigorous measures taken in China against COVID-19 and the beliefs people held about the virus in unaffected Western countries.

As early as January 2020, renowned epidemiologists like Gabriel Leung or Marc Lipsitch had highlighted the threat of a global pandemic (Wu et al., 2020). They announced that more than 40–70% of the world population could be infected within the end of the year. However, survey data collected in February 2020 during the early phases of the outbreak in France, Italy, the United Kingdom, and Switzerland showed that a large majority of citizens estimated their risk of catching the virus to be around 1% (Raude et al., 2020). Similar findings from the French International Market Research Group reported that only 47% of French citizens said they were worried about the virus, 53% of them did not wash their hands after taking public transportation, 75% continued to shake hands, and 91% still kissed their loved ones. At that very same time, SARS-CoV-2 had affected more than 160,000 people worldwide and killed 6,000. It then evolved within a few weeks from a rare, distant threat into an omnipresent health hazard affecting more than 2.900 000 people worldwide and killing 204,000 at the end of April 2020.

Importantly, data collected in Western countries during the peak of the COVID 19 pandemic provides direct evidence favoring the hypothesis that unrealistic optimism has played a role in the apparent discrepancy between official warnings and individual beliefs about the consequences of the pandemic for oneself: When getting infected and infecting others became frequent events as the number of cases and deaths sharply increased, citizens in the US, Europe and the United Kingdom estimated their probability of getting infected with the virus and of subsequently infecting others as lower for themselves than for someone else (Dolinski et al., 2020; Kuper-Smith et al., 2020).

These findings strikingly echo similar patterns observed during past epidemics such as severe acute respiratory syndrome coronavirus (SARS), influenza A virus subtype H1N1 (H1N1) and Middle East respiratory syndrome (MERS-CoV). During the 2009 influenza A virus subtype H1N1 pandemic for example, epidemiologists pointed out risks of infection ranging from 11 to 19%. However, the majority of people believed that they were unlikely to get infected and to infect others (Xu and Peng, 2015), and felt that the outbreak did not affect their daily lives (Lau et al., 2009). These findings strikingly echo the 2003 SARS outbreak (Brug et al., 2004; Jun et al., 2004).

## Alternative Explanations

During the early phases of a pandemic, when only a few cases are reported and case numbers vary greatly across the world, beliefs about risks of infection may have been shaped by other cognitive biases, too. In the context of an outbreak, beliefs are generated within a collective, massive, and chaotic set of evidence from local and global sources such as personal connections and the media (Rogers, 1975). Here, we argue that it is the degree to which people use this information to update their beliefs about the consequences of the pandemic that is asymmetrical, even though the beliefs in themselves differ much across individuals on whether they were under- or overestimations.

For example, people vary in how much they discount risks, as these risks are still temporarily and spatially distant. The variability in temporal and spatial discounting influences various beliefs including health-related ones, which are most relevant in the context of a pandemic (Peake, 2017; Lee et al., 2018).

Moreover, exponentially growth is often perceived in linear terms, which is also known as the exponential growth bias, and has recently been shown to have played a role in the misperception of the SARS-Cov-2 outbreak (Lammers et al., 2020).

In the context of a pandemic psychological myopia can provide another explanation for the slow adoption of cautious behavior such as social distancing. On the short term putting in place strict, mandatory measures of social distancing involves psychological, social, and economic costs (i.e., risks). Avoiding these short-term risks could come at the expense of the long-term health benefits of containing the outbreak (Thaler et al., 1997).

Moreover, it is possible that overgeneralization from past epidemics has influenced people's beliefs about the danger of SARS-Cov-2. Recent epidemics such as the 2003 SARS outbreak were overcome relatively easier. For example the virus rapidly spread across 30 countries, but was contained within about 6 months. While the WHO warned that the problem was not completely solved, and future outbreaks remained a possibility, any official transmissions were reported since the end of June 2003 (World Health Organization., 2003). This experience might have generated an underestimation of the dangers of the novel SARS-Cov-2 virus despite official warnings.

Another bias that could have played a role is linked to the fact that rare, adverse life events such as getting infected with a novel pathogen are more salient, and thus easier to retrieve from memory. This can have various effects on the beliefs about the likelihood of experiencing rare, adverse life events. More specifically, the coexistence hypothesis proposed by Barron and Yechiam predicts that people overestimate the likelihood of rare, adverse events (e.g., overestimate the probability of an uncertain loss of a large amount of money) and at the same time concurrently to their judgement underweight these events during decision-making (e.g., choose to loose the uncertain large amount rather than the certain loss of a small amount, despite equivalent expected value) (Barron and Yechiam, 2009). This hypothesis consolidates contradictory findings from judgement and decision-making from experience under both field and controlled laboratory conditions [see (Barron and Yechiam, 2009) for review]. In the context of a real-world problem such as a pandemic this line of research suggests that people overestimate the likelihood of short- and long-term consequences of the pandemic for themselves and for others, and at the same time underweight these likelihoods when adopting precautionary behaviors (Barron and Yechiam, 2009).

Prospect theory also offers another relevant explanation for beliefs generated in the context of a pandemic. It predicts that equivalent information has different effects on beliefs and precautionary behavior if it is framed either in terms of losses or of gains (Kahneman and Tversky, 1979; Tversky and Kahnemann, 1981). For example, loss-framed information, such as the number of death or negative societal consequences of an outbreak, has been shown to be not especially effective in promoting risk avoidance behaviors like hand washing, containment, or social isolation (Hameleers, 2020).

Taken together these alternative explanations stand in contrast to the idea that discrepancies between official warnings and individual assessments were due to optimistically biased beliefs. It is therefore important to distinguish if and how the beliefs in themselves and/or the update of these beliefs were biased during the early phases of the pandemic growth. For example, an initial overestimation of the risk could lead to a positive prediction error when being presented with case numbers that are lower than thought, especially in the beginning of a pandemic. On the behavioral level, such a positive prediction error could lead to considering less the need for social distancing (e.g., because the situation is less bad than initially thought), which may correspond to an underweighting of the initial probability judgment. On the other hand side, an initial underestimation is less used to update beliefs, and could lead to neglect on the behavioral level. Here we propose that the difference in the absolute update of beliefs, and not whether these beliefs were over- or underestimations, has influenced risk assessment during early phases of the SARS-Cov-2 outbreak characterized by great uncertainty of information nurturing such updating biases (Johnson and Fowler, 2011).

## Will The Coronavirus Epidemic Benefit From Human Cognitive Biases on The Long Term?

From an evolutionary perspective, biased beliefs represent a conundrum. On the one hand, unrealistic optimism appears to be a good coping mechanism in environments where the rewards are higher than the costs. Maintaining deflationary beliefs about risk is a kind of coping mechanism that allows individuals to protect themselves from the emotional manifestations associated with a threat, which is adaptive when the threat is distant and ambiguous. On the other hand, it can become dangerous when the costs are higher than the rewards. Our ancestral environment was strewn with great dangers and an error in judgment could quickly prove fatal. It is therefore plausible that the human brain is equipped with feedback mechanisms allowing is to adapt to situations in which an overly positive perspective would be harmful.

Interestingly, unrealistic optimism bias in belief updating diminishes when there is an immediate threat in the environment (Garrett et al., 2018). In other words, faced with an immediate threat, in a kind of risk adverse response humans devalue unfavorable information less, and use it more easily to update beliefs and form a mental model of the world. As COVID-19 is spreading rapidly throughout the world, the initially ambiguous and distant threat has become immediate and long lasting. Hence, we could expect that as infection risk increases and becomes ubiquitous, awareness improves, and potentially changes the ways in which beliefs are updated. Risk perception could thus become more accurate, as the unrealistic optimism bias in belief updating is dampened down. Consistent with this idea, a study carried out in the USA has shown that the perceived risk of getting COVID-19 increased dramatically over the course of 5 days after the WHO declared COVID-19 a pandemic (Wise et al., 2020). This study further found that engagement in preventive measures was strongly predicted by the perceived likelihood of personally being infected: the higher it was, the more people engaged in protective behaviors (Wise et al., 2020).

## Can Optimistic Biases be Leveraged In The Face of a Pandemic?

The challenge today is to maintain relatively high individual risk perceptions despite the prolongation of the crisis. It has been shown that a prolonged exposure to a threat increases feelings of familiarity, progressively reducing perceived risk (Chaudhary et al., 2004), as observed during the 2009 H1N1, and 2015 MERS-CoV epidemic (Cowling et al., 2010; Jang et al., 2020). Maintaining a consistent and relatively unbiased risk assessment is crucial for sustaining protective individual behavior, and will surely represent the main challenge for the coming months.

This effort should go along with more research in social and cognitive neuroscience. For example, future work in this field could shed light on how the evolution of distant to immediate health risks affects human beliefs about risk, and how the brain encodes the updating of these beliefs. It is also completely unknown how individual differences in temporal and spatial discounting moderate belief updating about risks of infection and of infecting others in the context of a pandemic and its evolution.

On another level, public health policies must maintain a good level of information and explanation, so that the population upholds appropriate beliefs about the risks associated with this crisis. For example, nudge theory applications propose using cognitive biases to influence individuals' behaviors, and may play a major role in guiding worldwide health strategies against the coronavirus (Thaler, 2018). These applications include a range of techniques such as scratch landmarks on the ground to promote social distancing, perfumed hydroalcoholic gels to encourage hand washing, or enhancing perceived risk by conveying information through popular peers together with expert advice. After falling victims to our cognitive biases, they may very well be what will save us.

As the previous health, geopolitical, and climatic cataclysms, this epidemic challenges neurocognitive models about economic power, industrial independence, or even the solidity of our healthcare systems. We call for more research to shed light on how humans around the world are adjusting their way of understanding the dangers associated with the pandemic. This is important to understand if and how this unprecedented health crisis in the 21st century opens the window into the human brain's remarkable capacities for adaptation, and to detect potential cognitive traps that can potentially hinder preparations for future waves of outbreaks and the adoption of early precautionary measures.

## Author Contributions

HB wrote the first draft of the manuscript. All authors contributed to the final text.

## Conflict of Interest

The authors declare that the research was conducted in the absence of any commercial or financial relationships that could be construed as a potential conflict of interest.
